# Rethinking clinical decision-making to improve clinical reasoning

**DOI:** 10.3389/fmed.2022.900543

**Published:** 2022-09-08

**Authors:** Salvatore Corrao, Christiano Argano

**Affiliations:** ^1^Department of Internal Medicine, National Relevance and High Specialization Hospital Trust ARNAS Civico, Palermo, Italy; ^2^Dipartimento di Promozione della Salute Materno Infantile, Medicina Interna e Specialistica di Eccellenza “G. D’Alessandro” (PROMISE), University of Palermo, Palermo, Italy

**Keywords:** clinical reasoning, metacognition, cognitive biases, Default Mode Network (DMN), clinical decision making

## Abstract

Improving clinical reasoning techniques is the right way to facilitate decision-making from prognostic, diagnostic, and therapeutic points of view. However, the process to do that is to fill knowledge gaps by studying and growing experience and knowing some cognitive aspects to raise the awareness of thinking mechanisms to avoid cognitive errors through correct educational training. This article examines clinical approaches and educational gaps in training medical students and young doctors. The authors explore the core elements of clinical reasoning, including metacognition, reasoning errors and cognitive biases, reasoning strategies, and ways to improve decision-making. The article addresses the dual-process theory of thought and the new Default Mode Network (DMN) theory. The reader may consider the article a first-level guide to deepen how to think and not what to think, knowing that this synthesis results from years of study and reasoning in clinical practice and educational settings.

## Introduction

Clinical reasoning is based on complex and multifaceted cognitive processes, and the level of cognition is perhaps the most relevant factor that impacts the physician’s clinical reasoning. These topics have inspired considerable interest in the last years (1, 2). According to Croskerry (3) and Croskerry and Norman (4), over 40 affective and cognitive biases may impact clinical reasoning. In addition, it should not be forgotten that both the processes and the subject matter are complex.

In medicine, there are thousands of known diagnoses, each with different complexity. Moreover, in line with Hammond’s view, a fundamental uncertainty will inevitably fail (5). Any mistake or failure in the diagnostic process leads to a delayed diagnosis, a misdiagnosis, or a missed diagnosis. The particular context in which a medical decision is made is highly relevant to the reasoning process and outcome (6).

More recently, there has been renewed interest in diagnostic reasoning, primarily diagnostic errors. Many researchers deepen inside the processes underpinning cognition, developing new universal reasoning and decision-making model: The Dual Process Theory.

This theory has a prompt implementation in medical decision-making and provides a comprehensive framework for understanding the gamma of theoretical approaches taken into consideration previously. This model has critical practical applications for medical decision-making and may be used as a model for teaching decision reasoning. Given this background, this manuscript must be considered a first-level guide to understanding how to think and not what to think, deepening clinical decision-making and providing tools for improving clinical reasoning.

## Too much attention to the tip of the iceberg

The New England Journal of Medicine has recently published a fascinating article (7) in the “Perspective” section, whereon we must all reflect on it. The title is “At baseline” (the basic condition). Dr. Bergl, from the Department of Medicine of the Medical College of Wisconsin (Milwaukee), raised that his trainees no longer wonder about the underlying pathology but are focused solely on solving the acute problem. He wrote that, for many internal medicine teams, the question is not whether but to what extent we should juggle the treatment of critical health problems of patients with care for their coexisting chronic conditions. Doctors are under high pressure to discharge, and then they move patients to the next stage of treatment without questioning the reason that decompensated the clinical condition. Suppose the chronic condition or baseline was not the fundamental goal of our performance. In that case, our juggling is highly inconsistent because we are working on an intermediate outcome curing only the decompensation phase of a disease. Dr. Bergl raises another essential matter. Perhaps equally disturbing, by adopting a collective “base” mentality, we unintentionally create a group of doctors who prioritize productivity rather than developing critical skills and curiosity. We agree that empathy and patience are two other crucial elements in the training process of future internists. Nevertheless, how much do we stimulate all these qualities? Perhaps are not all part of cultural backgrounds necessary for a correct patient approach, the proper clinical reasoning, and balanced communication skills?

On the other hand, a chronic baseline condition is not always the real reason that justifies acute hospitalization. The lack of a careful approach to the baseline and clinical reasoning focused on the patient leads to this superficiality. We are focusing too much on our students’ practical skills and the amount of knowledge to learn. On the other hand, we do not teach how to think and the cognitive mechanisms of clinical reasoning.

## Time to rethink the way of thinking and teaching courses

Back in 1910, John Dewey wrote in his book “How We Think” (8), “The aim of education should be to teach us rather how to think than what to think—rather improve our minds to enable us to think for ourselves than to load the memory with the thoughts of other men.”

Clinical reasoning concerns how to think and make the best decision-making process associated with the clinical practice (9). The core elements of clinical reasoning (10) can be summarized in:

1. Evidence-based skills,
2. Interpretation and use of diagnostic tests,
3. Understanding cognitive biases,
4. Human factors,
5. Metacognition (thinking about thinking), and
6. Patient-centered evidence-based medicine.

All these core elements are crucial for the best way of clinical reasoning. Each of them needs a correct learning path to be used in combination with developing the best thinking strategies (Table 1). Reasoning strategies allow us to combine and synthesize diverse data into one or more diagnostic hypotheses, make the complex trade-off between the benefits and risks of tests and treatments, and formulate plans for patient management (10).

**TABLE 1:** Set of some reasoning strategies (view the text for explanations).

Approaching uncommon clinical pictures
Gathering and assessing clinical data
Generating diagnostic hypotheses
Deciding on the appropriateness of diagnostic tests
Assessing test results
Assembling a coherent working diagnosis
Weighing the value of therapeutic approaches in the single patient

However, among the abovementioned core element of clinical reasoning, two are often missing in the learning paths of students and trainees: metacognition and understanding cognitive biases.

### Metacognition

We have to recall cognitive psychology, which investigates human thinking and describes how the human brain has two distinct mental processes that influence reasoning and decision-making. The first form of cognition is an ancient mechanism of thought shared with other animals where speed is more important than accuracy. In this case, thinking is characterized by a fast, intuitive way that uses pattern recognition and automated processes. The second one is a product of evolution, particularly in human beings, indicated by an analytical and hypothetical-deductive slow, controlled, but highly consuming way of thinking. Today, the psychology of thinking calls this idea “the dual-process theory of thought” (11–14). The Nobel Prize in Economic Sciences awardee Daniel Kahneman has extensively studied the dichotomy between the two modes of thought, calling them fast and slow thinking. “System 1” is fast, instinctive, and emotional; “System 2” is slower, more deliberative, and more logical (15). Different cerebral zones are involved: “System 1” includes the dorsomedial prefrontal cortex, the pregenual medial prefrontal cortex, and the ventromedial prefrontal cortex; “System 2” encompasses the dorsolateral prefrontal cortex. Glucose utilization is massive when System 2 is performing (16). System 1 is the leading way of thought used. None could live permanently in a deliberate, slow, effortful way. Driving a car, eating, and performing many activities over time become automatic and subconscious.

A recent brilliant review of Gronchi and Giovannelli (17) explores those things. Typically, when a mental effort is required for tasks requiring attention, every individual is subject to a phenomenon called “ego-depletion.” When forced to do something, each one has fewer cognitive resources available to activate slow thinking and thus is less able to exert self-control (18, 19). In the same way, much clinical decision-making becomes intuitive rather than analytical, a phenomenon strongly affected by individual differences (20, 21). Experimental evidence by functional magnetic resonance imaging and positron emission tomography studies supports that the “resting state” is spontaneously active during periods of “passivity” (22–25). The brain regions involved include the medial prefrontal cortex, the posterior cingulate cortex, the inferior parietal lobule, the lateral temporal cortex, the dorsal medial prefrontal cortex, and the hippocampal formation (26). Findings reporting high-metabolic activity in these regions at rest (27) constituted the first clear evidence of a cohesive default mode in the brain (28), leading to the widely acknowledged introduction of the Default Mode Network (DMN) concept. The DMN contains the medial prefrontal cortex, the posterior cingulate cortex, the inferior parietal lobule, the lateral temporal cortex, the dorsal medial prefrontal cortex, and the hippocampal formation. Lower activity levels characterize the DMN during goal-directed cognition and higher activity levels when an individual is awake and involved in the mental processes requiring low externally directed attention. All that is the neural basis of spontaneous cognition (26) that is responsible for thinking using internal representations. This paradigm is growing the idea of stimulus-independent thoughts (SITs), defined by Buckner et al. (26) as “thoughts about something other than events originating from the environment” that is covert and not directed toward the performance of a specific task. Very recently, the role of the DMN was highlighted in automatic behavior (the rapid selection of a response to a particular and predictable context) (29), as opposed to controlled decision making, suggesting that the DMN plays a role in the autopilot mode of brain functioning.

**FIGURE 1:**
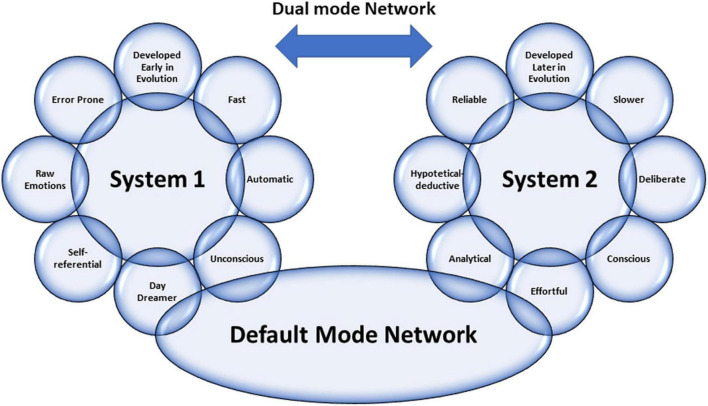
Graphical representation of the characteristics of Dual Mode Network, including the relationship between the two systems by Default Mode Network (view the text for explanations).

In light of these premises, everyone can pause to analyze what he is doing, improving self-control to avoid “ego-depletion.” Thus, one can actively switch between one type of thinking and the other. The ability to make this switch makes the physician more performing. In addition, a physician can be trained to understand the ways of thinking and which type of thinking is engaged in various situations. This way, experience and methodology knowledge can energize Systems 1 and 2 and how they interact, avoiding cognitive errors. Figure 1 summarizes all the concepts abovementioned about the Dual Mode Network and its relationship with the DMN.

Emotional intelligence is another crucial factor in boosting clinical reasoning for the best decision-making applied to a single patient. Emotional intelligence recognizes one’s emotions. Those others label different feelings appropriately and use emotional information to guide thinking and behavior, adjust emotions, and create empathy, adapt to environments, and achieve goals (30). According to the phenomenological account of Fuchs, bodily perception (proprioception) has a crucial role in understanding others (31). In this sense, the proprioceptive skills of a physician can help his empathic understanding become elementary for empathy and communication with the patient. In line with Fuchs’ view, empathic understanding encompasses a bodily resonance and mediates contextual knowledge about the patient. For medical education, empathy should help to relativize the singular experience, helping to prevent that own position becomes exclusive, bringing oneself out of the center of one’s own perspective.

### Reasoning errors and cognitive biases

Errors in reasoning play a significant role in diagnostic errors and may compromise patient safety and quality of care. A recently published review by Norman et al. (32) examined clinical reasoning errors and how to avoid them. To simplify this complex issue, almost five types of diagnostic errors can be recognized: no-fault errors, system errors, errors due to the knowledge gap, errors due to misinterpretation, and cognitive biases (9). Apart from the first type of error, which is due to unavoidable errors due to various factors, we want to mention cognitive biases. They may occur at any stage of the reasoning process and may be linked to intuition and analytical systems. The most frequent cognitive biases in medicine are anchoring, confirmation bias, premature closure, search satisficing, posterior probability error, outcome bias, and commission bias (33). Anchoring is characterized by latching onto a particular aspect at the initial consultation, and then one refuses to change one’s mind about the importance of the later stages of reasoning. Confirmation bias ignores the evidence against an initial diagnosis. Premature closure leads to a misleading diagnosis by stopping the diagnostic process before all the information has been gathered or verified. Search satisficing blinds other additional diagnoses once the first diagnosis is made posterior probability error shortcuts to the usual patient diagnosis for previously recognized clinical presentations. Outcome bias impinges on our desire for a particular outcome that alters our judgment (e.g., a surgeon blaming sepsis on pneumonia rather than an anastomotic leak). Finally, commission bias is the tendency toward action rather than inaction, assuming that only good can come from doing something (rather than “watching and waiting”). These biases are only representative of the other types, and biases often work together. For example, in overconfidence bias (the tendency to believe we know more than we do), too much faith is placed in opinion instead of gathered evidence. This bias can be augmented by the anchoring effect or availability bias (when things are at the forefront of your mind because you have seen several cases recently or have been studying that condition in particular), and finally by commission bias—with disastrous results.

### Novice vs. expert approaches

The reasoning strategies used by novices are different from those used by experts (34). Experts can usually gather beneficial information with highly effective problem-solving strategies. Heuristics are commonly, and most often successfully, used. The expert has a saved bank of illness scripts to compare and contrast the current case using more often type 1 thinking with much better results than the novice. Novices have little experience with their problems, do not have time to build a bank of illness scripts, and have no memories of previous similar cases and actions in such cases. Therefore, their mind search strategies will be weak, slow, and ponderous. Heuristics are poor and more often unsuccessful. They will consider a more comprehensive range of diagnostic possibilities and take longer to select approaches to discriminate among them. A novice needs specific knowledge and specific experience to become an expert. In our opinion, he also needs special training in the different ways of thinking. It is possible to study patterns, *per se* as well. It is, therefore, likely to guide the growth of knowledge for both fast thinking and slow one.

Moreover, learning by osmosis has traditionally been the method to move the novice toward expert capabilities by gradually gaining experience while observing experts’ reasoning. However, it seems likely that explicit teaching of clinical reasoning could make this process quicker and more effective. In this sense, an increased need for training and clinical knowledge along with the skill to apply the acquired knowledge is necessary. Students should learn disease pathophysiology, treatment concepts, and interdisciplinary team communication developing clinical decision-making through case-series-derived knowledge combining associative and procedural learning processes such as “Vienna Summer School on Oncology” (35).

Moreover, a refinement of the training of communicative skills is needed. Improving communication skills training for medical students and physicians should be the university’s primary goal. In fact, adequate communication leads to a correct diagnosis with 76% accuracy (36). The main challenge for students and physicians is the ability to respond to patients’ individual needs in an empathic and appreciated way. In this regard, it should be helpful to apply qualitative studies through the adoption of a semi-structured or structured interview using face-to-face in-depth interviews and e-learning platforms which can foster interdisciplinary learning by developing expertise for the clinical reasoning and decision-making in each area and integrating them. They could be effective tools to develop clinical reasoning and decision-making competencies and acquire effective communication skills to manage the relationship with patient (37–40).

## Clinical reasoning ways

Clinical reasoning is complex: it often requires different mental processes operating simultaneously during the same clinical encounter and other procedures for different situations. The dual-process theory describes how humans have two distinct approaches to decision-making (41). When one uses heuristics, fast-thinking (system 1) is used (42). However, complex cases need slow analytical thinking or both systems involved (15, 43, 44). Slow thinking can use different ways of reasoning: deductive, hypothetic-deductive, inductive, abductive, probabilistic, rule-based/categorical/deterministic, and causal reasoning (9). We think that abductive and causal reasoning need further explanation. Abductive reasoning is necessary when no deductive argument (from general assumption to particular conclusion) nor inductive (the opposite of deduction) may be claimed.

In the real world, we often face a situation where we have information and move backward to the likely cause. We ask ourselves, what is the most plausible answer? What theory best explains this information? Abduction is just a process of choosing the hypothesis that would best explain the available evidence. On the other hand, causal reasoning uses knowledge of medical sciences to provide additional diagnostic information. For example, in a patient with dyspnea, if considering heart failure as a casual diagnosis, a raised BNP would be expected, and a dilated vena cava yet. Other diagnostic possibilities must be considered in the absence of these confirmatory findings (e.g., pneumonia). Causal reasoning does not produce hypotheses but is typically used to confirm or refute theories generated using other reasoning strategies.

Hypothesis generation and modification using deduction, induction/abduction, rule-based, causal reasoning, or mental shortcuts (heuristics and rule of thumbs) is the cognitive process for making a diagnosis (9). Clinicians develop a hypothesis, which may be specific or general, relating a particular situation to knowledge and experience. This process is referred to as generating a differential diagnosis. The process we use to produce a differential diagnosis from memory is unclear. The hypotheses chosen may be based on likelihood but might also reflect the need to rule out the worst-case scenario, even if the probability should always be considered.

Given the complexity of the involved process, there are numerous causes for failure in clinical reasoning. These can occur in any reasoning and at any stage in the process (33). We must be aware of subconscious errors in our thinking processes. Cognitive biases are subconscious deviations in judgment leading to perceptual distortion, inaccurate assessment, and misleading interpretation. From an evolutionary point of view, they have developed because, often, speed is more important than accuracy. Biases occur due to information processing heuristics, the brain’s limited capacity to process information, social influence, and emotional and moral motivations.

Heuristics are mind shortcuts and are not all bad. They refer to experience-based techniques for decision-making. Sometimes they may lead to cognitive biases (see above). They are also essential for mental processes, expressed by expert intuition that plays a vital role in clinical practice. Intuition is a heuristic that derives from a natural and direct outgrowth of experiences that are unconsciously linked to form patterns. Pattern recognition is just a quick shortcut commonly used by experts. Alternatively, we can create patterns by studying differently and adequately in a notional way that accumulates information. The heuristic that rules out the worst-case scenario is a forcing mind function that commits the clinician to consider the worst possible illness that might explain a particular clinical presentation and take steps to ensure it has been effectively excluded. The heuristic that considers the least probable diagnoses is a helpful approach to uncommon clinical pictures and thinking about and searching for a rare unrecognized condition. Clinical guidelines, scores, and decision rules function as externally constructed heuristics, usually to ensure the best evidence for the diagnosis and treatment of patients.

Hence, heuristics are helpful mind shortcuts, but the exact mechanisms may lead to errors. Fast-and-frugal tree and take-the-best heuristic are two formal models for deciding on the uncertainty domain (45).

## Conclusion

In the recent times, clinicians have faced dramatic changes in the pattern of patients acutely admitted to hospital wards. Patients become older and older with comorbidities, rare diseases are frequent as a whole (46), new technologies are growing in a logarithmic way, and sustainability of the healthcare system is an increasingly important problem. In addition, uncommon clinical pictures represent a challenge for clinicians (47–50). In our opinion, it is time to claim clinical reasoning as a crucial way to deal with all complex matters. At first, we must ask ourselves if we have lost the teachings of ancient masters. Second, we have to rethink medical school courses and training ones. In this way, cognitive debiasing is needed to become a well-calibrated clinician. Fundamental tools are the comprehensive knowledge of nature and the extent of biases other than studying cognitive processes, including the interaction between fast and slow thinking. Cognitive debiasing requires the development of good mindware and the awareness that one debiasing strategy will not work for all biases. Finally, debiasing is generally a complicated process and requires lifelong maintenance.

We must remember that medicine is an art that operates in the field of science and must be able to cope with uncertainty. Managing uncertainty is the skill we have to develop against an excess of confidence that can lead to error. Sound clinical reasoning is directly linked to patient safety and quality of care.

## Data availability statement

The original contributions presented in this study are included in the article/supplementary material, further inquiries can be directed to the corresponding author.

## Author contributions

SC and CA drafted the work and revised it critically. Both authors have approved the submission of the manuscript.
